# Impact of Regional Trauma System Implementation on In-Hospital Mortality in Alahsa, Saudi Arabia: A Retrospective Comparative Cohort Study: Short title: Regional Trauma System and Mortality

**DOI:** 10.31661/gmj.v15i.4298

**Published:** 2026-08-13

**Authors:** Naif T. AlQahtani, Abdulelah Alanazi, Mujtaba M. Alkhamees, Faisal T. AlQahtani, Ahmed S. Alsaidlani, Ahmed F. Alharbi, Omer S. Alharbi, Mohammed Asiri, Abdulaziz Alrabiah

**Affiliations:** ^1^ Department of Emergency Medical Services, Prince Sultan bin Abdulaziz College for Emergency Medical Services, King Saud University, Riyadh, Saudi Arabia.; ^2^ Quality and patient safety director department, Alahsa Cluster, Ministry of Health, Saudi Arabia.; ^3^ King Fahad Hospital, Alahsa Cluster, Ministry of Health, Saudi Arabia.; ^4^ Health care assistance, Jazan region, Ministry of Defense, Saudi Arabia.; ^5^ Anesthesia Department, Armed Forces Hospital, Al-Kharj, Saudi Arabia.; ^6^ Trauma department, Trauma Path director, King Fahad Hospital, Alahsa Cluster, Ministry of Health, Saudi Arabia.; ^7^ Emergency Department, Medical City University Hospital, King Saud University, Riyadh, Saudi Arabia.

**Keywords:** Trauma Systems, Wounds, Injuries, Hospital Mortality, Injury Severity Score, Motor Vehicles, Vision 2030

## Abstract

**Background:**

Trauma remains a major cause of morbidity and mortality worldwide, and road traffic injuries represent a substantial public health burden in Saudi Arabia. Structured trauma systems may improve outcomes, but their regional impact in Saudi Arabia requires further evaluation. This study assessed in-hospital mortality and injury patterns before and after trauma system implementation in Alahsa, Saudi Arabia.

**Materials and Methods:**

This retrospective comparative cohort study was conducted at King Fahad Hospital, the Level I trauma center of the Alahsa Health Cluster. Trauma patients admitted during the pre-implementation period (2017–2018) and post-implementation period (2022–2023) were included if they had an Injury Severity Score (ISS) ≥15 and hospital stay >24 hours. Demographic data, injury mechanisms, anatomical injury sites, length of stay, and in-hospital mortality were analyzed. Bivariate analyses, multivariable logistic regression, Poisson regression sensitivity analysis, and interrupted time-series analysis were performed.

**Results:**

A total of 631 patients were included. Crude in-hospital mortality decreased from 19.0% before implementation to 16.3% after implementation, but this difference was not statistically significant (P=0.390). After adjustment for age, mechanism of injury, and ISS, the post-implementation period was not independently associated with mortality (AOR=0.97; 95% CI: 0.45–2.10; P=0.945). ISS and age were the strongest independent predictors of mortality. Significant shifts were observed in injury mechanisms and anatomical injury patterns, including reduced face and neck injuries and increased thoracic injuries.

**Conclusion:**

Trauma system implementation in Alahsa was associated with numerically lower crude mortality and changes in injury patterns, but mortality reduction was not statistically significant after case-mix adjustment. These findings support continued trauma registry development, pediatric trauma pathways, and subspecialty service expansion.

## Introduction

Trauma remains one of the leading global health challenges, accounting for an estimated five million deaths annually and surpassing the combined mortality of malaria, tuberculosis, and HIV/AIDS [1]. Beyond its human cost, trauma contributes to profound social and economic burdens, particularly in low- and middle-income countries where road traffic injuries and workplace accidents disproportionately affect younger, economically active populations [2, 3].

To address this challenge, many nations have invested in structured trauma systems, integrated networks of pre-hospital, hospital, and rehabilitative services designed to ensure timely, coordinated, and specialized care for severely injured patients [4]. A trauma system typically comprises three tiers of service provision: Level I centers, which function as comprehensive hubs for trauma leadership, advanced surgical capability, research, and education; Level II units, which deliver acute resuscitation and life-saving surgical interventions; and Level III centers, which stabilize patients before transfer to higher levels of care [5, 6]. Evidence accumulated over the past five decades demonstrates that such systems significantly improve survival and functional outcomes. In the United Kingdom, the centralization of trauma services led to a substantial increase in case volume, a reduction in secondary transfers, and measurable gains in recovery outcomes [7]. Similarly, the establishment of regional trauma systems in the Netherlands, Canada, and the United States has been associated with reductions in mortality ranging from 15-25% [8, 9, 10]. In the United Arab Emirates, the development of a structured trauma network in Al Ain reduced in-hospital mortality among trauma patients by more than half [11]. These findings underscore the transformative potential of coordinated trauma care, particularly in regions with high injury burdens.

In the Kingdom of Saudi Arabia, trauma has remained the foremost cause of death and disability over the past four decades, with road traffic collisions representing the predominant mechanism of severe injury [6]. While national prevention initiatives, such as mandatory seatbelt use, automated speed enforcement, and occupational safety programs, have yielded improvements in selected cities [12, 13], trauma continues to exact a heavy toll. Importantly, the Kingdom’s Vision 2030 reform agenda explicitly recognize trauma system development and emergency care strengthening as strategic priorities to reduce preventable mortality and improve health system performance [14].

Within this framework, the Alahsa Health Cluster has progressively restructured its trauma services. The cluster, serving approximately 1.3 million residents with more than 2,000 hospital beds and 65 primary care centers, includes a single Level I trauma center and a network of Level II and III hospitals [15]. This configuration provides an opportunity to evaluate, in a defined regional context, whether system-level investments translate into measurable improvements in patient outcomes.

This study investigates the effectiveness of trauma system implementation in the Alahsa region of Saudi Arabia by comparing demographic patterns, injury mechanisms, and clinical outcomes, including mortality, before and after the restructuring of services.

## Materials and Methods

### Study Design and Setting

We conducted a retrospective comparative cohort study at King Fahad Hospital, the designated Level I trauma center within the Alahsa Health Cluster, Saudi Arabia. The cluster serves approximately 1.3 million residents across 11 hospitals and 65 primary care centers. King Fahad Hospital provides comprehensive trauma care and functions as the referral hub for major injuries in the region. Study Population and Eligibility Criteria All trauma patients admitted to King Fahad Hospital during two predefined study periods were eligible for inclusion: January 2017 December 2018 (pre-implementation of the regional trauma system) and January 2022 December 2023 (post-implementation). Data were collected manually from patient medical records for both study periods (2017-2018 and 2022-2023) using a standardized data collection form, and there is no change after electronic health-record implementation. Inclusion criteria were: patients of any age hospitalized for more than 24 hours, and patients with an Injury Severity Score (ISS) ≥ 15, and the AIS version is 2015, and ISS coded was by main author. Patients dead on arrival or transferred out within 24 hours were excluded to avoid incomplete outcome assessment.

### Data Collection and Variables

Data were extracted from the trauma registry of King Fahad Hospital, which prospectively records clinical and demographic information on all admitted patients. The dataset included age, sex, and nationality, the latter dichotomized as Saudi or non-Saudi. Mechanism of injury was classified into motor vehicle accidents, falls, burns, or penetrating trauma. The anatomical location of injury was categorized as head, thorax, abdomen or pelvis, upper extremities, lower extremities, face or neck, and external. Severity was assessed using the ISS. Clinical outcomes of interest were in-hospital mortality and length of stay expressed in days.

### Outcome Measures

The principal outcome of interest was all-cause in-hospital mortality. Secondary outcomes were the distribution of injury mechanisms, anatomical patterns of injury, and hospital length of stay.

### Statistical Analysis

All analyses were performed using IBM SPSS Statistics version 27.0 (Armonk, NY, USA) and RStudio version 2025.09.2+418. Descriptive statistics were used to summarize the data, with quantitative variables expressed as means ± standard deviations or medians with interquartile ranges (IQR) based on the Kolmogorov–Smirnov test for normality. Categorical variables were presented as absolute frequencies and percentages. To evaluate the impact of the trauma system implementation, the following inferential methods were applied: Bivariate Analysis: Comparisons between the pre-implementation (2017-2018) and post-implementation (2022-2023) periods for categorical variables were conducted using the Pearson Chi-square test. A simple logistic regression model was applied to calculate the unadjusted Odds Ratio (OR) and 95% Confidence Intervals (CI) for the primary mortality outcome, and the absolute risk difference was also reported. The Mann–Whitney U test was employed for continuous variables with a non-normal distribution, such as hospital length of stay. Primary Adjusted Model, Multivariable Logistic Regression: Because in-hospital mortality is a binary outcome, multivariable logistic regression was used as the primary adjusted model. It estimated the adjusted odds ratio (AOR) with 95% CI for the study period while adjusting for age, mechanism of injury, and ISS, with burns as the reference mechanism. ISS was available for the analyzed cases and was retained in this primary model, and a complete-case approach was applied. Baseline physiological parameters (blood pressure, heart rate, respiratory rate) were not consistently archived in the electronic registry during the pre-implementation period (2017-2018) and were therefore excluded to ensure a consistent comparison across both cohorts. Poisson Regression (sensitivity analysis): As a sensitivity analysis, a Poisson regression model estimated the Incidence Rate Ratio (IRR) for in-hospital mortality, adjusting for study period, patient age, and mechanism of injury. This approach helps confirm that observed changes in outcomes are not merely artifacts of shifts in patient demographics or injury types. Interrupted Time Series (ITS) Analysis: A longitudinal evaluation was performed to assess shifts in mortality trends over the study period. This quasi-experimental design was selected as the most robust method for evaluating the temporal impact of system-level health interventions. An ITS model was constructed using a Poisson distribution, incorporating an offset variable (log of total monthly cases) to adjust for fluctuations in monthly patient volume. This ensured that changes in mortality rates were evaluated independently of changes in regional trauma case volume. Statistical significance was defined as a two-tailed P-value < 0.05, and exact p values with effect sizes and confidence intervals are reported.

### Ethical Considerations

The study was conducted in accordance with the principles of the Declaration of Helsinki and received approval from the Institutional Review Board of the Alahsa Health Cluster, King Fahad Hospital Hofuf (IRB No. H-05-HS-065; Log No. 41-EP-2024; approval date 27 August 2024; category: expedited approval). Because of the retrospective design and the use of anonymized data, the requirement for individual patient informed consent was waived by the IRB.

## Results

**Table:** Table1. Demographic and clinical characteristics of the study population

Variables	Number (%)
Age (years)	
<15	101 (16.0)
15–30	221 (35.0)
31–45	200 (31.7)
46–60	69 (10.9)
>60	40 (6.3)
Study period	
2017–2018	368 (58.3)
2022-2023	263 (41.7)
Sex	
Male	551 (87.3)
Female	80 (12.7)
Nationality	
Saudi	462 (73.2)
Non-Saudi	169 (26.8)
Mechanism of injury	
Burn	22 (3.5)
Fall	24 (3.8)
Motor vehicle accident	581 (92.0)
Penetrating trauma	4 (0.6)
In-hospital mortality	
No	518 (82.1)
Yes	113 (17.9)

**Table:** Table2. Comparison of demographic and clinical characteristics between pre- and post-implementation periods

Variables	Years 2017-2018	Years 2022-2023	P-value	
Age (Years) ^a^	Less than 15	64 (63.4%)	37 (36.6%)	0.313
	15-30	120 (54.3%)	101 (45.7%)	
	31-45	124 (62.0%)	76 (38.0%)	
	46-60	36 (52.2%)	33 (47.8%)	
	Above 60	24 (60.0%)	16 (40.0%)	
Gender ^a^	Male	315 (57.2%)	236 (42.8%)	0.124
	Female	53 (66.3%)	27 (33.8%)	
Nationality ^a^	Saudi	302 (65.4%)	160 (34.6%)	<0.001*
	Non-Saudi	66 (39.1%)	103 (60.9%)	
Mortality ^a^	70 (19.0%)	43 (16.3%)	0.390	
Length of Stay (LOS) ^b^	Mean ± SD	13.7 ± 62.8	11.2 ± 29.5	0.296
	Median (IQR)	5.0 (2.0 – 11.0)	5.0 (2.0 – 9.0)	
Injury Severity Score (ISS) ^b^	Mean ± SD	30.5 ± 15.7	28.8 ± 14.2	0.141
	Median (IQR)	25.0 (20 - 38)	25.0 (17-36)	

a: values are n (%), P-value by Pearson Chi-square test. b: values are mean ± SD or median (IQR), P-value by Mann-Whitney test. * Significant at P ≤ 0.01.

**Table:** Table3. Comparison of the distribution of mechanisms of injury between pre- and post-implementation periods

Mechanism	Years 2017-2018	Years 2022-2023	P-value
MVA	334 (90.8%)	238 (90.5%)	
Falls	8 (2.2%)	16 (6.1%)	0.006*
Burns	14 (3.8%)	8 (3.0%)	
Penetrating trauma	12 (3.3%)	1 (0.4%)	

Values are n (%); P-value by Pearson Chi-square test. ^*^ Significant at P ≤ 0.01.

**Table:** Table4. Comparison of the location of injury between pre- and post-implementation periods

Location		Years 2017-2018	Years 2022-2023	P-value
Head	Yes	207 (56.3%)	134 (51.0%)	0.188
	No	161 (43.8%)	129 (49.0%)	
Abdominal and pelvis	Yes	92 (25.0%)	89 (33.8%)	0.015*
	No	276 (75.0%)	174 (66.2%)	
Upper extremities	Yes	49 (13.3%)	34 (12.9%)	0.887
	No	319 (86.7%)	229 (87.1%)	
Lower extremities	Yes	70 (19.0%)	57 (21.7%)	0.413
	No	298 (81.0%)	206 (78.3%)	
Face and neck	Yes	65 (17.7%)	24 (9.1%)	0.002*
	No	303 (82.3%)	239 (90.9%)	
Thoracic	Yes	89 (24.2%)	119 (45.2%)	< 0.001*
	No	279 (75.8%)	144 (54.8%)	
External	Yes	19 (5.2%)	16 (5.7%)	0.767
	No	349 (94.8%)	248 (94.3%)	

Values are n (%); P-value by Pearson Chi-square test. ^*^ Significant at P ≤ 0.01.

**Table:** Table5. Comparison of parameters in the child patients (≤ 14) between pre- and post-implementation periods

Variables		Years 2017-2018	Years 2022-2023	P-value
Mortality a	Yes	8 (13.8%)	4 (11.8%)	0.525
	No	50 (86.2%)	30 (88.2%)	
Mechanism of injury b	Burn	1 (1.7%)	2 (5.9%)	0.003**
	Fall	0 (0.0%)	3 (8.8%)	
	MVA	57 (98.3%)	29 (85.3%)	
Location of occurrence of injury				
Head b	Yes	45 (77.6%)	26 (76.5%)	0.902
	No	13 (22.4%)	8 (23.5%)	
Abdominal and pelvis b	Yes	12 (20.7%)	10 (29.4%)	0.334
	No	46 (79.3%)	24 (70.6%)	
Face and neck a	Yes	11 (19.0%)	1 (2.9%)	0.024*
	No	47 (81.0%)	33 (97.1%)	
Thoracic a	Yes	7 (12.1%)	15 (44.1%)	< 0.001**
	No	51 (87.9%)	19 (55.9%)	
Length of Stay (LOS) c	Mean ± SD	7.14 ± 9.89	6.78 ± 10.0	0.735
	Median (IQR)	3.0 (1.25 – 8.0)	3.0 (1.0 – 7.0)	

a: Pearson Chi-square test; b: Fisher’s exact test; c: Mann-Whitney test. Values are n (%) unless stated. MVA: Motor Vehicle Accidents^**^ P ≤ 0.01 and ^*^ P ≤ 0.05 are Significant.

**Table:** Table6. Comparison of parameters in adult patients (aged > 14) between the pre- and post-implementation periods

Variables		Years 2017-2018	Years 2022-2023	P-value
Mortality ^a^	Yes	61 (21.1%)	37 (17.3%)	0.285
	No	228 (78.9%)	177 (82.7%)	
Mechanisms of injury	MVA ^a^	268 (92.7%)	195 (91.1%)	0.123
	Falls ^a^	8 (2.8%)	13 (6.1%)	
	Burn ^a^	13 (4.5%)	6 (2.8%)	
Location of occurrence of injury				
Head ^a^	Yes	147 (50.9%)	100 (46.7%)	0.359
	No	142 (49.1%)	114 (53.3%)	
Abdominal and pelvis ^a^	Yes	76 (26.3%)	75 (35.0%)	0.034*
	No	213 (73.7%)	139 (65.0%)	
Upper extremities ^a^	Yes	44 (15.2%)	31 (14.5%)	0.818
	No	245 (84.8%)	183 (85.5%)	
Lower extremities ^a^	Yes	59 (20.4%)	53 (24.8%)	0.246
	No	230 (79.6%)	161 (75.2%)	
Face and neck ^a^	Yes	51 (17.6%)	20 (9.3%)	0.008**
	No	238 (82.4%)	194 (90.7%)	
Thoracic ^a^	Yes	78 (27.0%)	99 (46.3%)	< 0.001**
	No	211 (73.0%)	115 (53.7%)	
External ^a^	Yes	14 (4.8%)	13 (6.1%)	0.545
	No	275 (95.2%)	201 (93.9%)	
Length of Stay (LOS) ^b^	Mean ± SD	15.5 ± 71.9	11.1 ± 30.1	0.198
	Median (IQR)	5.0 (2.0- 12.0)	5.0 (2.0 – 10.0)	

a: Pearson Chi-square test; b: Mann-Whitney test. Values are n (%) unless stated. MVA: Motor Vehicle Accidents. ** P ≤ 0.01 and * P ≤ 0.05 are Significant.

**Table:** Table7. Poisson Regression Model (sensitivity analysis) for factors associated with in-hospital mortality

Variable	Coefficient (β)	SE	P-value	IRR (95% CI)
Intercept	-0.372	0.397	0.35	-
Group (post-implementation)	-0.131	0.205	0.521	0.877 [0.584-1.30]
Age	0.009	0.005	0.076	1.009 [0.999-1.02]
Mechanism: Burn (reference)				1.0
Mechanism: Fall	-0.136	0.424	0.749	0.873 [0.391-2.10]
Mechanism: MVA	-2.07	0.367	< 0.001***	0.126 [0.064-0.277]
Mechanism: Penetrating Trauma	0.113	0.604	0.852	1.119 [0.302-3.46]

SE= Standard Error; IRR = Incidence Rate Ratio; CI = Confidence Interval. Reference for Groups is Pre-implementation (2017–2018). MVA: Motor Vehicle Accidents.

**Table:** Table8. Interrupted Time Series (ITS) Analysis of Trauma System Impact on Mortality Rates

Variable	Coefficient (β)	SE	Change (%)	IRR (95% CI)	P-value
Baseline Level (Intercept)	-1.424	0.225	-	0.241 [0.154-0.375]	< 0.001
Pre-intervention Trend (Time)	-0.019	0.017	-2	0.980 [0.949-1.013]	0.233
Level Shift (Intervention)	-0.269	0.401	-23	0.764 [0.344-1.696]	0.503
Trend Change (Time After)	0.049	0.029	5	1.05 [0.992-1.111]	0.087

SE= Standard Error; IRR = Incidence Rate Ratio; CI = Confidence Interval. The model includes an offset for log(total cases) to adjust for monthly fluctuations in patient volume.

**Table:** Table9. Multivariable Logistic Regression Analysis for In-Hospital Mortality

Predictors	OR [95% CI]	Wald	P-value
Age	1.04 [1.02-1.07]	15.57	< 0.001**
Group (post-implementation)	0.973 [0.450-2.10]	0.005	0.945
Injury Severity Score (ISS)	1.22 [1.17-1.27]	84.96	< 0.001**
Mechanism of Injury: Burn (reference)	1.00		
Mechanism of Injury (Fall)	0.980 [0.068-14.0]	0.000	0.988
Mechanism of Injury (MVA)	0.114 [0.022-0.595]	6.63	0.010*

For Mechanism of Injury, the reference category is burn. For the group, the reference category is pre-implementation. OR is the adjusted odds ratio; CI is the confidence interval. The model demonstrated a strong fit (Nagelkerke R² = 0.776) and an overall predictive accuracy of 95.1%. MVA: Motor Vehicle Accidents. ** P ≤ 0.01 and * P ≤ 0.05 are Significant.

**Figure-1:**
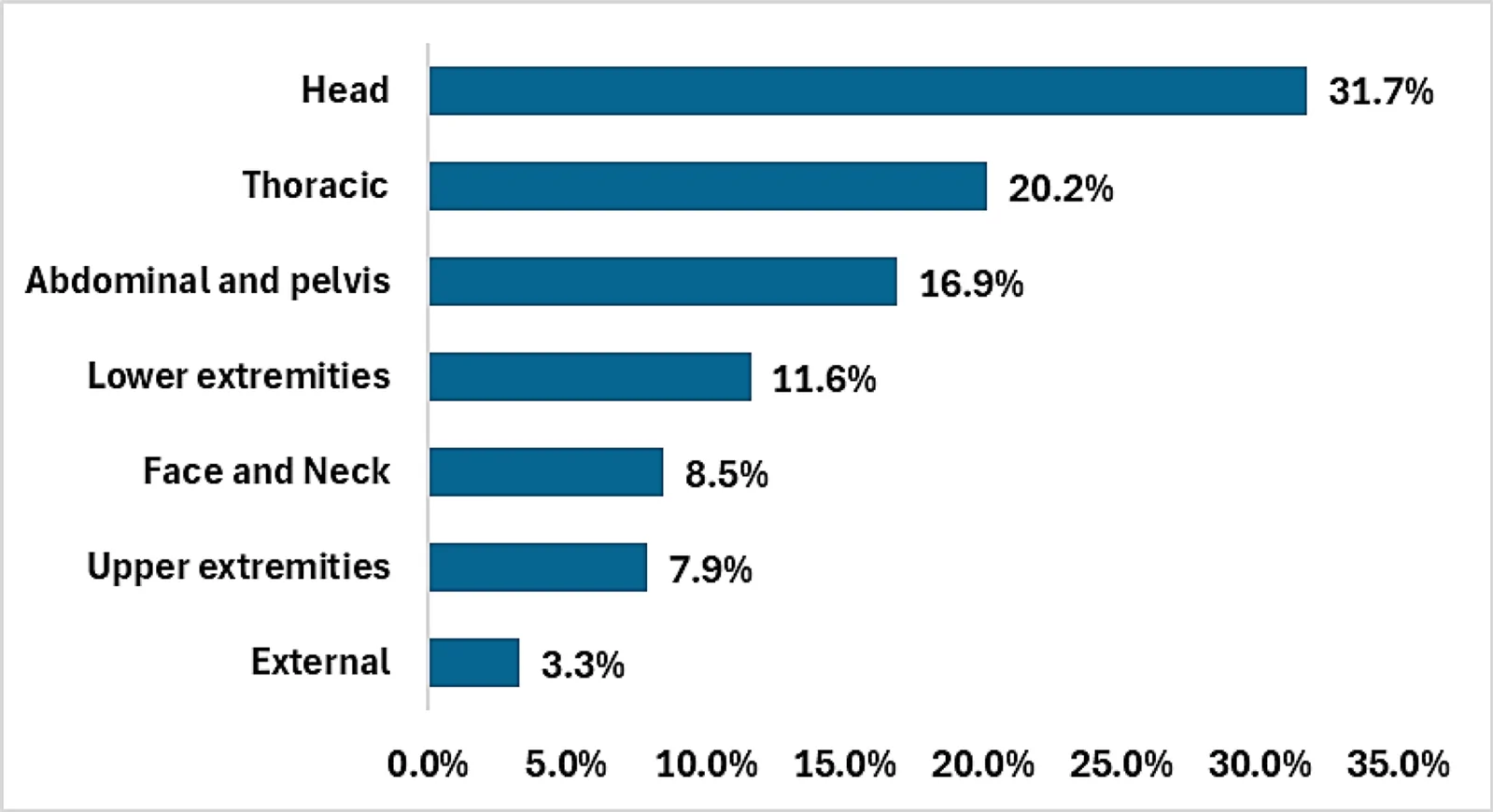
Figure 1. Distribution of injury locations among the study sample.

**Figure-2:**
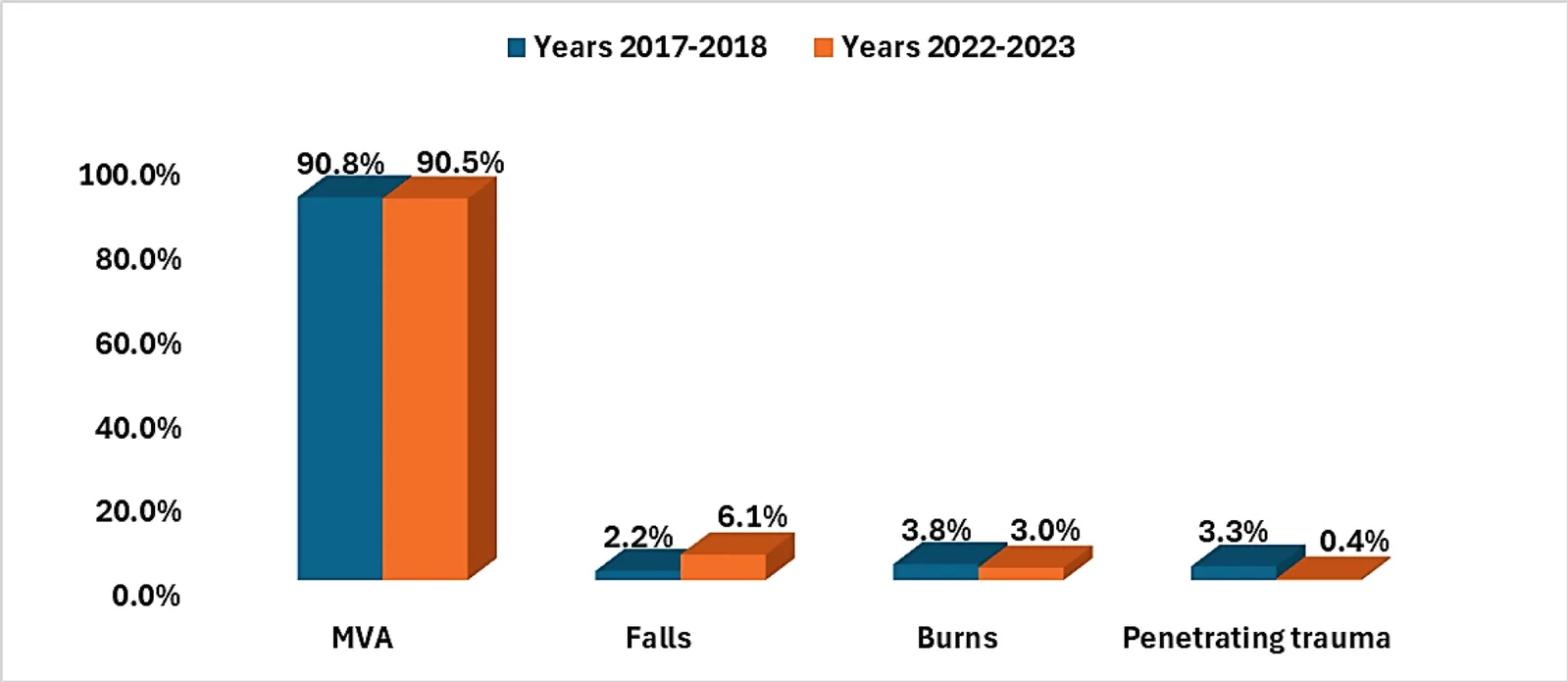
Figure 2. Mechanisms of Injury. Pre- vs. Post-Implementation Period.

**Figure-3:**
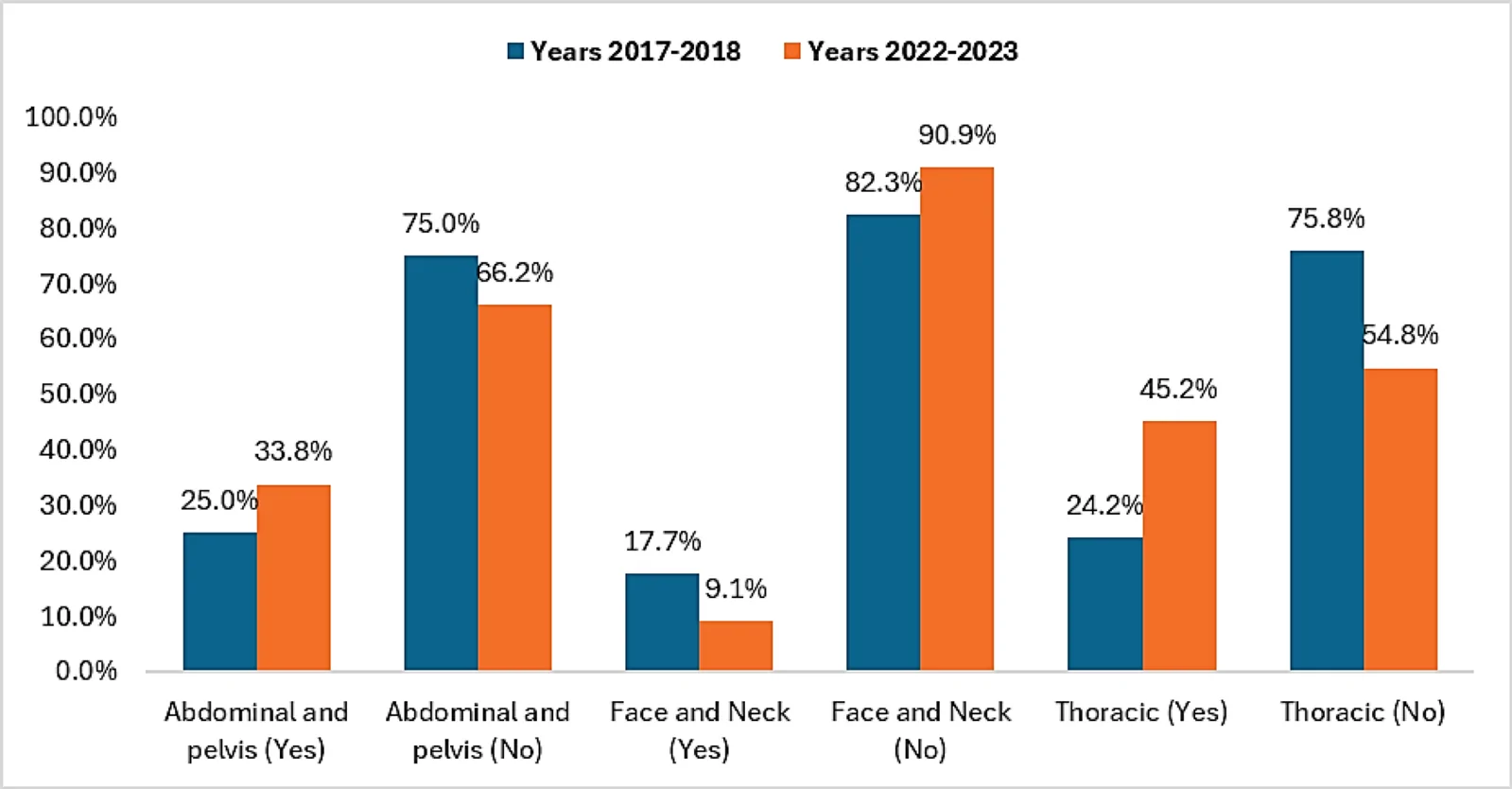
Figure 3. Comparison of abdominal and pelvic, face & neck, and thoracic injuries in the pre- and post-implementation periods

**Figure-4:**
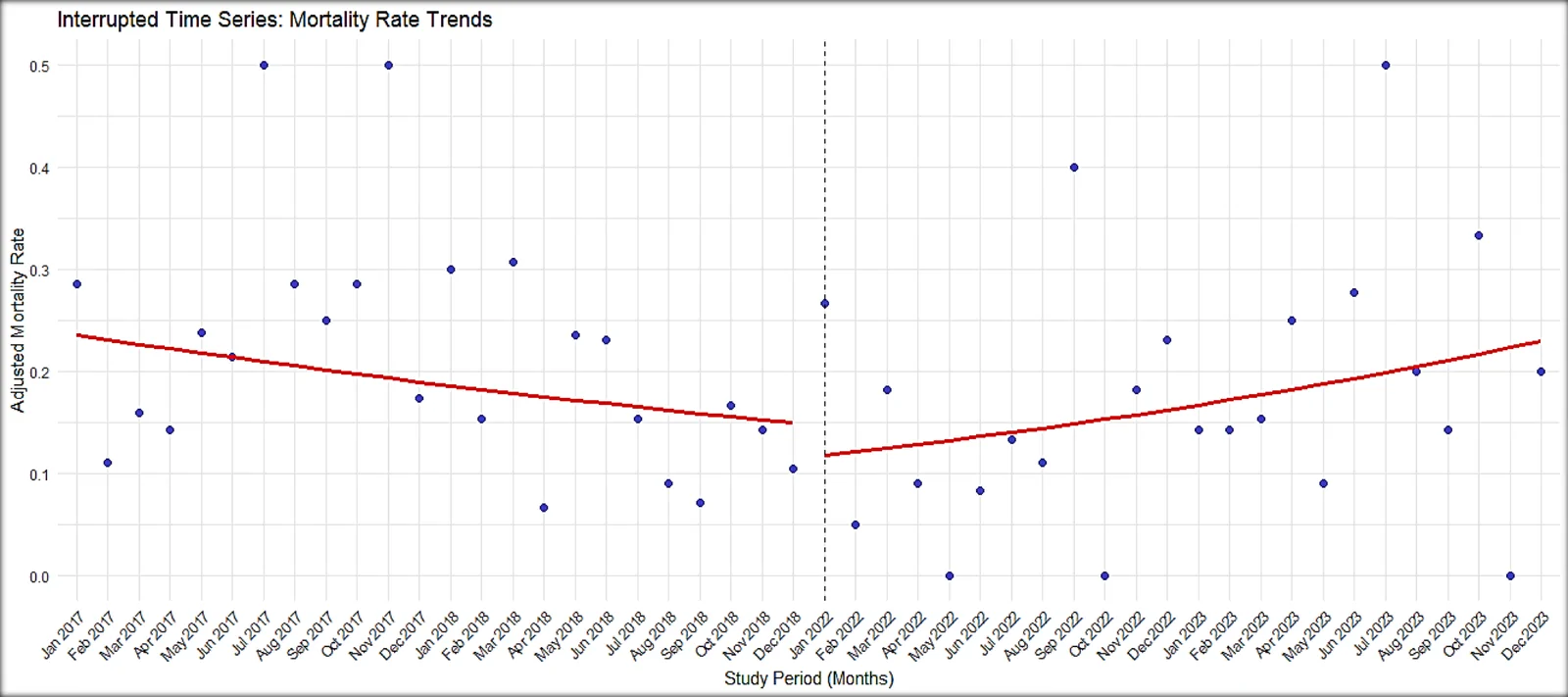
Figure 4. Interrupted Time Series (ITS) Analysis of Monthly Mortality Rates. The vertical dashed line indicates the system implementation point (January 2022).

### Study Population

A total of 631 trauma patients met the inclusion criteria across both study periods. The mean age of the cohort was 31.8 years (SD 16.6; range 1–90 years). Young adults were the most represented age group, with 35.0% of patients aged 15–30 years and 31.7% aged 31-45 years. Children accounted for 16.0% of admissions, whereas patients older than 60 years represented only 6.3% of the sample. The majority of cases were male (87.3%) and of Saudi nationality (73.2%). Motor vehicle accidents were the predominant mechanism of injury, responsible for over 90% of admissions, while burns, falls, and penetrating injuries constituted a minority of cases. The overall in-hospital mortality rate was 17.9%. Table1 summarizes the demographic and clinical characteristics of the study population. Figure-1 illustrates the anatomical distribution of injuries, with head injuries being most frequent (31.7%), followed by thoracic (20.2%) and abdominal or pelvic trauma (16.9%). Comparison of Outcomes Before and After Trauma System Implementation Of the included patients, 368 were admitted in the pre-implementation period (2017–2018) and 263 in the post-implementation period (2022–2023). Table2 presents the comparative distribution of demographic and clinical variables. The age distribution was not statistically significant between the two groups (P=0.313). The proportion of Saudi patients declined significantly after implementation, from 65.4% to 34.6%, with a corresponding increase in non-Saudi patients (P<0.001). In terms of crude mortality rates, in-hospital mortality was 19.0% (70/368) pre-implementation and 16.3% (43/263) post-implementation. However, this 2.7% absolute reduction was not statistically significant (Pearson χ² P=0.390), and the unadjusted OR for mortality in the post-implementation period was 0.83 (95% CI 0.55–1.26), a confidence interval that includes 1.0. No significant differences were observed in gender distribution, in hospital length of stay (P=0.296), or in Injury Severity Score (P=0.141).

### Changes in Mechanisms of Injury

Table3 demonstrates a statistically significant shift in the overall distribution of injury mechanisms between the pre- and post-implementation periods (P=0.006). In the pre-implementation period, motor vehicle accidents (MVA) represented the overwhelming majority of admissions (90.8%), decreasing slightly to 90.5% in the post-implementation period. However, the most notable changes were observed in the other injury categories. The percentage of penetrating trauma cases admitted to the Level I center showed a significant drop from 3.3% in the pre-implementation period to 0.4% in the post-implementation period. Conversely, the proportion of falls admitted increased sharply from 2.2% to 6.1% between the two periods. Burns showed a slight decrease (from 3.8% to 3.0%). Figure-2 depicts the trends in motor vehicle accidents and penetrating trauma across the two study periods.

### Changes in Anatomical Distribution of Injuries

The anatomical distribution of injuries is shown in Table4. Following trauma system implementation, head injuries decreased in frequency (from 56.3% to 51.0%), but this change was not statistically significant (P=0.188). A significant change was observed in other anatomical areas: face and neck injuries fell from 17.7% to 9.1% (P=0.002); thoracic injuries increased from 24.2% to 45.2% (P < 0.001); and abdominal and pelvic injuries increased from 25.0% to 33.8% (P=0.015). No significant differences were observed in upper extremities (P=0.887), lower extremities (P=0.413), or external injuries (P=0.767). Figure-3 illustrates these shifts. Thoracic injuries increased significantly in the post-implementation period (P<0.001), whereas face and neck injuries decreased significantly (P=0.002). Abdominal and pelvic injuries also increased significantly (P=0.015). No meaningful changes were observed for upper extremity, lower extremity, or external injuries. Subgroup Analysis: Pediatric Patients This subgroup analysis is exploratory and hypothesis-generating owing to small numbers and sparse cells. Table5 summarizes outcomes for children aged 14 years or younger. In this subgroup, the overall distribution of injury mechanisms demonstrated a significant shift in the post-implementation period (P=0.003). Specifically, the proportion of motor vehicle accidents (MVA) decreased from 98.3% to 85.3%, while admissions due to falls and burns increased. Regarding anatomical location, a significant reduction was observed in face and neck injuries, which fell from 19.0% to 2.9% (P=0.024). Conversely, thoracic injuries increased in frequency post-implementation (P < 0.001). The reduction in head injuries (from 77.6% to 76.5%) was not statistically significant (P=0.902). Mortality (P=0.525) did not differ significantly between the two periods, and the mean hospital length of stay showed a non-significant change (P=0.735).

Subgroup Analysis: Adult Patients As with the paediatric analysis, adult subgroup findings are exploratory. Among adults (> 14 years), the implementation of the trauma system was not associated with a statistically significant reduction in mortality (P=0.285). The overall distribution of injury mechanisms did not change significantly between the two periods (P=0.123). Significant shifts were observed in the anatomical location of injuries: face and neck injuries fell from 17.6% to 9.3% (P=0.008); thoracic injuries increased from 27.0% to 46.3% (P < 0.001); and abdominal and pelvic injuries increased from 26.3% to 35.0% (P=0.034). The reduction in head injuries (from 50.9% to 46.7%) was not statistically significant (P=0.359). No significant differences were observed in upper or lower extremities. Although the difference in length of stay was not statistically significant (P=0.198), the mean LOS for adults decreased from 15.5 to 11.1 days in the post-implementation period (Table6). As a sensitivity analysis, a multivariable Poisson regression model was performed (Table7). After adjusting for age and mechanism of injury, the Incidence Rate Ratio (IRR) for the post-implementation period was 0.877 (P=0.521), i.e., not an independent change. With burns as the reference category, motor vehicle accidents carried a lower rate of death than burns (IRR 0.126; P < 0.001); this should not be read as MVA being a predictor of higher mortality. Estimates for burns and penetrating trauma are unstable because of small numbers. To evaluate the longitudinal impact of the trauma system implementation, an Interrupted Time Series (ITS) analysis was conducted using a Poisson regression model adjusted for monthly case volume (offset) (Table8). The pre-intervention period (2017-2018) showed a stable mortality trend (P=0.233). Upon system implementation in January 2022, an immediate 23.6% reduction in the mortality rate was observed (IRR = 0.764), although this level shift did not reach statistical significance (P=0.503). Notably, the post-intervention trend change approached statistical significance (P=0.087), suggesting a transition in the underlying mortality dynamics following the restructuring. By adjusting for total case volume, this analysis confirms that observed shifts in mortality rates are independent of fluctuations in hospital admissions, addressing concerns regarding variations in regional trauma volumes. However, Because the series consists of two separated blocks bracketing the excluded 2019-2021 interval, this analysis is interpreted cautiously and is consistent with rather than confirmation of stability of mortality after accounting for case volume. The Interrupted Time Series (ITS) analysis further elucidated the temporal impact of the trauma system (Figure-4). Following the implementation in January 2022, an immediate shift in the mortality rate was observed (Level change IRR = 0.764), followed by a notable transition in the post-intervention trend (P=0.087).

### Confounder Control and Multivariable Results

To overcome the limitations of bivariate comparisons and account for potential confounding variables, the analysis progressed to a multivariable stage. The primary adjusted model was the multivariable logistic regression (Table9), with the Poisson model reported only as a sensitivity analysis. By incorporating age, mechanism of injury, and ISS, the models controlled for shifts in patient demographics and injury severity between the two periods, providing a more accurate estimate of the study period’s independent association with mortality.

### Multivariable Analysis and Case-Mix Adjustment

To rigorously evaluate the independent impact of the trauma system implementation, a multivariable logistic regression model was performed. This model incorporated study period (pre- vs. post-implementation), patient age, mechanism of injury, and the Injury Severity Score (ISS) to adjust for case-mix variation. The model exhibited strong predictive capacity, explaining 77.6% of the variance in mortality (Nagelkerke R² = 0.776) with an overall classification accuracy of 95.1% (Table9). The analysis revealed that mortality was driven by baseline clinical parameters rather than the study period. The Injury Severity Score was the strongest independent predictor of mortality; each one-point increase in ISS raised the adjusted odds of death by 22% (AOR = 1.22; 95% CI: 1.17–1.27; P<0.001). Advancing age also increased the odds of mortality (AOR = 1.04; 95% CI: 1.02–1.07; P<0.001). Crucially, after adjusting for baseline injury severity and age, the post-implementation period was not an independent predictor of mortality (AOR = 0.97; 95% CI: 0.45–2.10; P=0.945).

## Discussion

In this retrospective comparison, crude in-hospital mortality was numerically lower after implementation of a structured trauma system in the Alahsa Health Cluster (19.0% vs 16.3%), but this difference was not statistically significant and the study period was not independently associated with mortality after adjustment for age, mechanism, and ISS. Alongside these outcomes, notable changes were observed in both the mechanisms and anatomical distribution of injuries. These findings align with international evidence that trauma system development is associated with altered patterns of injury presentation, underscoring the clinical and policy relevance of sustained investment in system-level reforms.

The crude mortality rates were 19.0% pre-implementation and 16.3% post-implementation; the previously reported figures of “61.9%” and “38.1%” were the shares of the 113 total deaths falling in each period (70/113 and 43/113), not mortality rates, and have been corrected. A crude reduction of this direction is broadly comparable to outcomes reported elsewhere. In the United Arab Emirates, Alao et al. documented a 56% reduction in mortality following the establishment of a structured trauma network, while in the Netherlands van Aert GJ et al. observed a 16% mortality reduction linked to regional trauma system reorganization [8, 11]. Our findings also mirror the results of a systematic review and meta-analysis by Moore et al., which concluded that trauma system structure is consistently associated with decreased injury-related mortality across diverse settings [2].

The subgroup patterns differed by age but should be regarded as exploratory. Neither adult (P=0.285) nor paediatric (P=0.525) mortality changed significantly. Adult patients exhibited declines in facial or neck injuries, whereas pediatric patients experienced a shift in injury patterns, with decreases in facial trauma and an increase in thoracic injuries. These differences suggest that the effectiveness of trauma systems may be mediated by age-specific factors, including mechanisms of injury, physiology, and pre-hospital response. Comparable findings have been reported in high-income countries, where pediatric trauma care often requires specialized pathways to achieve equivalent survival gains [16]. The increase in thoracic trauma among children deserves closer monitoring, as it may reflect changing injury mechanisms or diagnostic practices rather than a true rise in incidence.

Motor vehicle accidents remained the dominant mechanism in both periods rather than declining. Although external factors such as stricter enforcement of traffic regulations, the introduction of automated detection cameras, and broader adoption of seatbelt use have been shown to reduce road traffic injuries in Saudi Arabia, these operate as parallel co-interventions that our design cannot disentangle from system effects [17, 18]. This is consistent with global trends, where integrated trauma systems also influence injury epidemiology through better data collection, surveillance, and prevention strategies [19].

Anatomical distribution of injuries also shifted in the post-implementation period, with a significant reduction in facial trauma and a non-significant reduction in head trauma. This parallels findings from the UK, where Cole et al. documented improved neurological outcomes after trauma system centralization [5]. The persistence of thoracic and abdominal trauma highlights the need for continued expansion of subspecialty services such as cardiothoracic and hepatobiliary surgery across regional hospitals.

Our results have important implications for Saudi Arabia’s Vision 2030 agenda. The Alahsa trauma system illustrates how system-level investments coincide with shifts in injury patterns within a relatively short period. The shift in patient demographics, with a growing proportion of non-Saudi patients in the post-implementation period, also highlights the importance of ensuring equitable access for a diverse population. These results resonate with recent evaluations of Gulf Cooperation Council trauma infrastructure, which emphasized variability in system maturity and the need for region-specific strategies to achieve uniform standards of care [20].

From a policy perspective, the findings support continued development of trauma registries and quality improvement programs as central pillars of trauma care. In particular, strengthening pediatric trauma pathways, enhancing subspecialty coverage for thoracic and abdominal injuries, and integrating public health interventions targeting road safety could amplify the benefits already achieved. Furthermore, the observed changes underscore the importance of trauma system evaluation at regular intervals, as both injury patterns and health system performance evolve in response to broader social and regulatory changes.

From a broader clinical and policy standpoint, these findings provide several important directions for strengthening trauma care in Saudi Arabia and comparable health systems. First, the results strongly support the integration of trauma registries into national health information systems. Reliable, real-time data collection enables monitoring of injury trends, benchmarking of outcomes, and rapid identification of system gaps. The Alahsa experience suggests that routine auditing of trauma care pathways should become an essential component of continuous quality improvement.

Second, further emphasis should be placed on pediatric trauma care. Although children in this study demonstrated significant reductions in facial injuries, mortality did not decline, indicating a need for specialized pediatric trauma protocols, equipment, and training. Developing pediatric-specific trauma teams and referral networks could help to ensure that any survival gains observed in adult populations are extended to children.

Third, the persistence of thoracic and abdominal trauma highlights the importance of broadening subspecialty surgical coverage. Investment in training and retaining cardiothoracic and hepatobiliary surgeons within regional hospitals would reduce delays in definitive management and further improve outcomes. This aligns with international evidence that subspecialty density is directly associated with survival in major trauma.

Fourth, system-level reforms should be complemented by robust injury prevention strategies. Road traffic injuries remain the leading cause of trauma admissions [21, 22]. Expanding preventive initiatives, such as stricter enforcement of mobile phone use and speeding laws, community education campaigns, and collaboration with transport authorities, could yield additional gains.

Finally, the findings underscore the need for sustained political and financial commitment to trauma system development as part of the broader Vision 2030 agenda. Trauma systems should not be regarded solely as hospital-based interventions but as integrated, multi-sectoral programs that link pre-hospital, hospital, rehabilitation, and public health domains. Embedding trauma system development within the Kingdom’s strategic health transformation program would ensure its long-term sustainability and enable Saudi Arabia to position itself as a regional leader in trauma care innovation.

## Limitations

This study has several limitations that should be acknowledged. First, the analysis was confined to a single Level I trauma center within the Alahsa Health Cluster, which may limit the generalizability of the findings to other regions of Saudi Arabia with different resources, referral pathways, and patient demographics. Second, the retrospective design relies on the accuracy and completeness of registry data, which is subject to potential underreporting or miscoding of variables such as mechanism and anatomical site of injury. Third, although we compared two well-defined time periods, unmeasured confounders, including parallel public health interventions, enforcement of road safety legislation, or broader changes in healthcare delivery, may have contributed to the observed shifts in injury patterns.

Fourth, the adjusted model included only age, mechanism, study period, and ISS; other important prognostic variables including sex, transfer status, mode of arrival, pre-hospital and emergency-department times, Glasgow Coma Scale, systolic blood pressure, respiratory rate, comorbidities, ICU admission, operative intervention, transfusion, neurosurgical availability, and detailed anatomical injury pattern—were not consistently available and could not be adjusted for, leaving residual confounding.

Finally, while the Injury Severity Score (ISS) remains a widely accepted measure of trauma severity, it does not capture all relevant dimensions of injury burden, particularly functional outcomes or long-term disability, which were not assessed in this study, and the study did not include a detailed analysis of pre-hospital care or inter-hospital transfer practices.

Given the observational and retrospective nature of this study, the observed changes should be interpreted as associations rather than direct causation, as unmeasured confounding factors may have influenced the outcomes. Despite these limitations, the study provides valuable evidence of the impact of trauma system implementation within a large regional cluster and highlights key directions for further multicenter and prospective research in Saudi Arabia.

## Conclusion

Implementation of a structured trauma system in the Alahsa Health Cluster was accompanied by a numerically lower crude mortality that did not reach statistical significance, together with meaningful shifts in injury patterns. Multivariable analysis revealed that mortality was primarily driven by variations in patient age and baseline injury severity (ISS) rather than a standalone systemic effect. These findings emphasize the critical importance of rigorous case-mix adjustment in trauma systems evaluation and suggest the potential value of system-level investments in trauma care to ensure stable survival rates across varying injury profiles. Strengthening pediatric trauma pathways, expanding subspecialty coverage, and embedding trauma registries into national health reforms will be essential to consolidating these gains. The experience in Alahsa may provide valuable insights for the ongoing development of trauma systems in other regions of Saudi Arabia. However, further multicenter studies and broader evaluations are needed to determine the generalizability of these findings and to inform national trauma system implementation across Saudi Arabia in alignment with Vision 2030.

## Conflict of Interest

The authors declare no conflict of interest.

## AI Disclosure Statement

The authors declare that no generative artificial intelligence tools were used in the design of the study, data collection, data analysis, interpretation of the findings, or preparation of this manuscript.

## References

[R1] World Health Organization World health statistics 2025: monitoring health for the SDGs,
Sustainable Development Goals. Geneva: World Health Organization; 2025..

[R2] Moore L, Champion H, Tardif PA, Kuimi BL, O’Reilly G, Leppaniemi A, et al (2018). Impact of trauma-system structure on injury outcomes: a
systematic review and meta-analysis. World J Surg.

[R3] Kwon J, Lee M, Song S, Kim S, Moon J, Huh Y, et al Impact of qualified trauma center implementation on mortality
from severe trauma in Korea: a retrospective cohort study. J Korean Med Sci. 2025;40(32):e196..

[R4] Patel VR, Rozycki G, Jopling J, Subramanian M, Kent A, Manukyan M, et al (2024). Association between geospatial access to trauma center care and
motor vehicle crash mortality in the United States. J Trauma Acute Care Surg.

[R5] Alharbi RJ, Shrestha S, Lewis V, Miller C (2021). The effectiveness of trauma care systems at different stages of
development in reducing mortality: a systematic review and meta-analysis. World J Emerg Surg.

[R6] Chowdhury S, Mok D, Leenen L (2022). Development of the Saudi Arabian trauma system. J Med Life.

[R7] Cole E (2022). The national major trauma system within the United Kingdom:
inclusive regionalized networks of care. Emerg Crit Care Med.

[R8] van Aert GJ, van der Laan L, Boonman-de Winter LJ, Berende CA, de Groot HG, van Hensbroek PB, et al Effect of the COVID-19 pandemic during the first lockdown in the
Netherlands on the number of trauma-related admissions, trauma severity and
treatment: the results of a retrospective cohort study in a level 2 trauma
centre. BMJ Open. 2021;11(2):e045015..

[R9] Morris RS, Karam BS, Murphy PB, Jenkins P, Milia DJ, Hemmila MR, et al Field-triage, hospital-triage and triage-assessment: a literature
review of the current phases of adult trauma triage. J Trauma Acute Care Surg. 2021;90(6):e138-45..

[R10] Alao DO, Cevik AA, Eid HO, Jummani Z, Abu-Zidan FM (2020). Trauma system developments reduce mortality in hospitalized
trauma patients in Al-Ain City, United Arab Emirates, despite increased
severity of injury. World J Emerg Surg.

[R11] Alghnam S, Towhari J, Alkelya M, Binahmad A, Bell TM (2018). The effectiveness of introducing detection cameras on compliance
with mobile phone and seatbelt laws: a before-after study among drivers in
Riyadh, Saudi Arabia. Inj Epidemiol.

[R12] Alenezi EZ, AlQahtani AM, Althunayan SF, Alanazi AS, Aldosari AO, Alharbi AM, et al Prevalence and determinants of road traffic accidents in Saudi
Arabia: a systematic review. Cureus. 2023;15(12):e51205..

[R13] Mani ZA, Goniewicz K (2024). Transforming healthcare in Saudi Arabia: a comprehensive
evaluation of Vision 2030’s impact. Sustainability.

[R14] Al-Ahsa Health Cluster Al-Ahsa Health Cluster. Saudi Arabia: Health Holding Company; 2025..

[R15] Razzak JA, Bhatti J, Wright K, Nyirenda M, Tahir MR, Hyder AA (2022). Improvement in trauma care for road traffic injuries: an
assessment of the effect on mortality in low-income and middle-income
countries. Lancet.

[R16] Alobaidallah AM, Alqahtany A, Maniruzzaman KM (2025). Safety effectiveness of automated traffic enforcement systems: a
critical analysis of existing challenges and solutions. Future Transp.

[R17] Rahman MM, Islam MK, Al-Shayeb A, Arifuzzaman M (2022). Towards sustainable road safety in Saudi Arabia: exploring
traffic accident causes associated with driving behavior using a Bayesian
belief network. Sustainability.

[R18] Goldman S, Cohen-Manheim I, Radomislensky I, Savitsky B, Bahouth H, Bar A, et al (2024). Demographic and injury trends for car crash casualties
hospitalized in level I trauma centers over two decades: data from the
National Trauma Registry. Isr J Health Policy Res.

[R19] Khan L, Karim A, Bey H, Alzaid SN, Al-Qadhi HA, Alabboudi YH, et al (2025). Evaluating Gulf Cooperation Council trauma care infrastructure: a
scoping review of key components and gaps. World J Surg.

[R20] Goniewicz K, Burkle FM, Khorram-Manesh A (2025). Transforming global public health: climate collaboration,
political challenges, and systemic change. J Infect Public Health.

[R21] De Biasi A, Krupa JM, Rzotkiewicz A, McGarrell E, Circo G, Liebler J (2023). Insights from law enforcement personnel during the COVID-19
pandemic: a focus on violence reduction and prevention initiatives and
firearm violence. Police Pract Res.

[R22] AlShahwan N, Aldeligan SH, Althunayan ST, Alkodari A, Bin Manee M, Albassam FA, et al (2025). Outcomes of traumatic liver injuries at a level-one tertiary
trauma center in Saudi Arabia: a 10-year experience. Life.

